# How to diagnose dry anophthalmic socket syndrome (DASS) in the clinical routine

**DOI:** 10.1007/s00417-023-06074-5

**Published:** 2023-04-27

**Authors:** Alexander C. Rokohl, Marc Trester, Keith R. Pine, Ludwig M. Heindl

**Affiliations:** 1 Department of Ophthalmology, University of Cologne, Faculty of Medicine and University Hospital of Cologne, Kerpener Straße 62, 50937 Cologne, Germany; 2 Center for Integrated Oncology (CIO) Aachen-Bonn-Cologne-Düsseldorf, Cologne, Germany; 3 Trester-Institute for Ocular Prosthetics and Artificial Eyes, Cologne, Germany; 4 School of Optometry and Vision Science, University of Auckland, Auckland, New Zealand

**Keywords:** Dry anophthalmic socket syndrome, Tear film osmolarity, Matrix metalloproteinase 9, Enucleation, Evisceration, Conjunctival inflammation, Anophthalmic socket surface inflammation


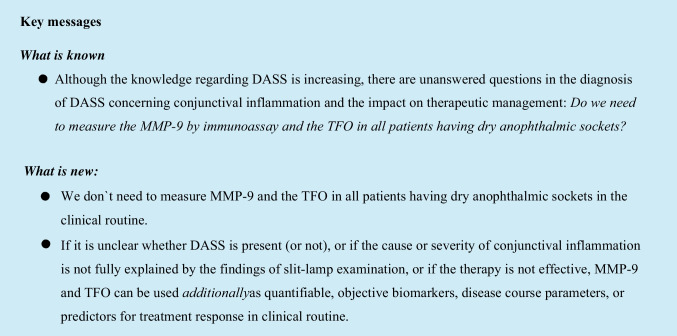


The editorial *How to analyze conjunctival inflammation in dry anophthalmic socket syndrome (DASS)?* by our highly esteemed colleague and friend Frederic Mouriaux was of great interest to us [1]. Fortunately, the knowledge regarding DASS is increasing and the pathophysiology of DASS is beginning to be understood [2–4]. Conjunctival inflammation is one of the key factors in DASS, most likely even the cardinal factor and should therefore be targeted for successful treatment [1]. However, Frederic Mouriaux leaves two unanswered questions in the diagnosis of DASS concerning conjunctival inflammation and the impact on therapeutic management [1]: *Do we need to measure the MMP-9 by immunoassay in all patients having anophthalmic sockets? And do we need to measure the TFO in all patients having anophthalmic sockets?*

When evaluating the subjective symptoms of DASS, standardized questionnaires including OSDI, DEQ-5, or SANDE should be used, separately for the anophthalmic socket and the healthy fellow eye routinely [1–4]. While DEQ-5 and SANDE can be used for healthy eyes and anophthalmic sockets the same way, in OSDI all vision-related questions (i.e., questions according to driving, watching TV) have to be classified as “not answered” for the anophthalmic side [2–4]. The total OSDI scores have then to be calculated based on the following formula (for each side separately): OSDI score = [(sum of scores for all questions answered) × 100] / [(total number of questions answered) × 4] [2–4].

For a comprehensive evaluation, a standardized clinical basis examination should include slit-lamp examination with special regard to conjunctival inflammation, anterior and posterior blepharitis, eyelid position, blinking rate, and lagophthalmos [2–4]. Measuring the tear film break-up time could also be helpful. However, since there are no validated absolute values for break-up time measurements in prosthetic eye wearers and it is sometimes, especially in patients having lagophthalmos, difficult to measure, break-up time should only be used as an individual follow-up parameter. The fit and surface condition of the prosthesis should also be checked [2–5]. In most of anophthalmic patients, these clinical basic examinations should be sufficient to diagnose or exclude DASS.

However, in some cases, further diagnostics are recommended, especially if it is unclear whether DASS is present, or if the cause or severity of conjunctival inflammation is not fully explained by the findings of slit-lamp examination, or if the therapy is not effective. In these cases, further examinations can be useful [2–4]. Since the use of Schirmer tests in anophthalmic sockets is not evidence-based, TFO measurements and MMP-9 point-of-care immunoassays can be used *additionally* as quantifiable, objective biomarkers, disease course parameters, or predictors for treatment response in clinical routine [2–4]. Imaging of the meibomian glands, quantifying the tear meniscus and goblet cells, examining the lacrimal drainage system, and evaluating the bacterial flora might also be useful [2–4]. Of course, most of these additional examinations cannot be performed easily in every ophthalmologist’s practice. Therefore, the consultation of specialized centers ideally having integrated care for anophthalmic patients is recommended in these cases.

We therefore conclude and are able to respond to the open questions *Do we need to measure the MMP-9 by immunoassay in all patients having anophthalmic sockets? And do we need to measure the TFO in all patients having anophthalmic sockets?*

No, we do not need it in the clinical routine. However, these measurements can be helpful in selected unclear cases.
